# Animal Allergens and Their Presence in the Environment

**DOI:** 10.3389/fimmu.2014.00076

**Published:** 2014-03-03

**Authors:** Eva Zahradnik, Monika Raulf

**Affiliations:** ^1^Institute for Prevention and Occupational Medicine of the German Social Accident Insurance, Institute of the Ruhr-Universität Bochum (IPA), Bochum, Germany

**Keywords:** animal allergens, allergen exposure, environmental monitoring, cats, dogs, rodents, cattle, horses

## Abstract

Exposure to animal allergens is a major risk factor for sensitization and allergic diseases. Besides mites and cockroaches, the most important animal allergens are derived from mammals. Cat and dog allergies affect the general population; whereas, allergies to rodents or cattle is an occupational problem. Exposure to animal allergens is not limited to direct contact to animals. Based on their aerodynamic properties, mammalian allergens easily become airborne, attach to clothing and hair, and can be spread from one environment to another. For example, the major cat allergen Fel d 1 was frequently found in homes without pets and in public buildings, including schools, day-care centers, and hospitals. Allergen concentrations in a particular environment showed high variability depending on numerous factors. Assessment of allergen exposure levels is a stepwise process that involves dust collection, allergen quantification, and data analysis. Whereas a number of different dust sampling strategies are used, ELISA assays have prevailed in the last years as the standard technique for quantification of allergen concentrations. This review focuses on allergens arising from domestic, farm, and laboratory animals and describes the ubiquity of mammalian allergens in the human environment. It includes an overview of exposure assessment studies carried out in different indoor settings (homes, schools, workplaces) using numerous sampling and analytical methods and summarizes significant factors influencing exposure levels. However, methodological differences among studies have contributed to the variability of the findings and make comparisons between studies difficult. Therefore, a general standardization of methods is needed and recommended.

## Introduction

Exposure to animal allergens is a major risk factor for the development of sensitization and allergic diseases such as asthma, allergic rhinitis/conjunctivitis, and atopic dermatitis (1, 2). Generally, intensive contact with any animal, including diverse arthropods, reptiles, birds, and mammals, can induce allergic reactions (3, 4). Apart from ubiquitous mites and cockroaches, the most frequent allergies developed are those to domesticated mammals with fur that are typically kept as pets or farm animals (1, 5). Contact with animals arises via many different occupations and activities. Cats, dogs, guinea pigs, hamsters, and rabbits are all very popular pets in industrialized countries, where the percentage of pet ownership continues to increase (6). Horses, whose use has decreased in agriculture, are today widely owned for recreational riding and show activities. Besides pigs, cows are the most common farm animals used for dairy and meat production. Another important source of occupational animal allergies is the handling of laboratory animals (7). Rodents, especially mice and rats, are kept in large numbers in research facilities of universities and pharmaceutical industries. In addition to these rodents housed in laboratories or occasionally kept as pets, mice, and rats can infest human urban and agricultural environments, where they find food supplies and have few predators.

During the past few decades, the distribution of various mammalian allergens has been extensively studied. Based on this research, it can be stated that animal allergens are present ubiquitously in the human environment, even though concentrations differ considerably. Numerous studies of animal allergen exposure levels in different locations and geographical regions have been published, with variable results that have been attributed to the use of different study designs (e.g., kind and number of samples, exposure grouping, data analysis). A mandatory requirement to assess allergen exposure is the availability of a reliable assay for allergen quantification. Therefore, this article focuses on well-characterized mammalian allergens derived from cats, dogs, mice, rats, cows, and horses for which very sensitive and specific ELISA assays have been validated. Some of these assays are currently commercially available (Indoor Biotechnologies, Charlottesville, VA, USA). Although, several allergens from other mammals (e.g., rabbit, guinea pig, ferret, hamster, chinchilla) have also been identified [summarized by Díaz-Perales et al. (6)], no assays for these species have been developed or published so far.

As a background, a short overview of characteristics of major allergens and sensitization rates in different population groups are presented in this report. The main focus of this review is a summary of exposure assessment studies conducted in recent years. Reported allergen levels and relevant factors associated with concentration differences are described for different exposure settings. Examples of studies were selected based on the fact that they were carried out in different environments (homes, workplaces, public buildings) using various dust sampling methods. Recommendations with regard to standardization of methods are being made for future research.

## Common Features of Animal Allergens Influencing Their Ubiquity

Inhalant allergens derived from mammals comprise a large and complex group. With the exception of the cat allergen Fel d 1, the majority of all animal major allergens belong to the lipocalin protein family (8). Typically, lipocalins from mammals are small extracellular proteins composed of approximately 150–170 amino acids with a molecular mass of about 20 kDa. Lipocalins share common biological functions that are predominantly related to the transport of small hydrophobic molecules such as steroids, odorants, and pheromones. Although, the overall amino acid identity between lipocalins is low (usually between 20 and 30%), this protein family is characterized by conserved sequence motifs and a common tertiary structure. Albumins represent another large protein family containing several respiratory allergens. They are components of serum and regulate osmotic pressure of blood. Compared to lipocalins, albumins are of minor allergic importance, but they are often responsible for allergic cross-reactions between animal dander of different species due to the high sequence identity among family members (about 80%) (9, 10). A partial cross-reactivity also exists between several lipocalins; however, the clinical relevance of this feature needs to be assessed (11).

Animal allergens are mainly produced in the liver or secretory glands and localized in animal skin and body fluids, such as urine, saliva, blood, milk, and sweat. These proteins adhere to fur and other surfaces. The allergens can be efficiently dispersed into the environment as animals shed hair and dander, and secrete and excrete fluids. Indoors, the allergens accumulate primarily in different textiles, including carpets, upholstery, mattresses, and curtains where they are detectable for a long time, even after removal of the animal (12–14). In addition, the aerodynamic properties of animal allergens influence their environmental distribution and human exposure. Especially, lipocalins tend to be carried on a diverse range of small dust particles, from <1 to 20 μm. Some proportion of allergens (<5 μm) can stay suspended in the air for extended periods of time (15). In contrast, mite allergens are primarily associated with large-sized (>20 μm) dust particles that settle rapidly (16). Based on their aerodynamic characteristics, animal allergens can be transferred to environments that were never occupied by the animals, such as public buildings, including schools, day-care centers, hospitals, and offices (17). Although, the concentrations of the allergens are low in these environments, they may be high enough to cause symptoms in sensitized individuals (18, 19).

There is strong evidence that clothing is the primary transfer mechanism of allergens. Significantly higher allergen levels have been found in dust collected from pet owners’ clothes than from the clothes of non-pet owners (20, 21). Furthermore, allergen levels have been shown to be dependent on clothing type and washing frequency (22). Egmar et al. (23) studied the accumulation of animal allergens in furniture stores by comparing the dust from factory-new mattresses to used ones. Allergen concentration correlated to the period of time that the mattresses were used by customers. In addition to clothing, human hair is also an important vehicle for transfer, and thus a source of animal allergens (24, 25).

## Measurement of Allergen Exposure

Measuring airborne allergens is necessary to detect allergen sources, to assess the relationship between allergen exposure, sensitization, and symptoms and to generate preventive measures. Allergen exposure assessment comprises two essential procedures: collection of dust samples and quantification of allergen levels. For both steps, a large variety of sampling strategies and analytical tests are available. However, the different methods used have made it difficult to compare the results among the different studies. Although, a general standardization would be preferred, in practice the variations cannot be avoided. The choice of sampling method is often influenced by the size of the study, available budget, practical performance, aerodynamics of the relevant allergens, and relevance to the personal exposure. Each method has advantages and disadvantages depending on the aims and technical limitations of the investigation (26).

### Dust sampling

*Reservoir* or *settled dust* sampling by vacuuming of surfaces is the most common method used for determining domestic exposure, e.g., pet allergens at home. It is inexpensive, easy, and fast to perform and therefore, widely applied in large-scale studies. Dust is collected on filters or in nylon bags mounted in special sampling devices that are commercially available (27). Several surfaces can be vacuumed, including floors (smooth or carpeted), beds (mattress or bedding), and furniture (sofas, chairs, desks). Apart from the collector and surface type, differences occur in the power of vacuum cleaner, size of the area sampled, sampling time, and sampling location (living room, bedroom, or kitchen). Collected dust may be sieved by some researchers to separate coarse particles. All the variations described influence the amount and composition of dust, and ultimately the results of the allergen analysis. Results are expressed as allergen per unit weight or per square meter, and although significant relationships have been shown between allergen levels in reservoir dust and allergic diseases, the relevance to personal exposure is still questionable. Many collected dust particles never become airborne and are therefore never inhaled.

*Airborne dust* sampling using pumps is commonly used in occupational settings, for example to measure mouse allergens in laboratories. According to the aerodynamic properties of animal allergens, airborne levels might be more suitable for defining exposure to pets. This method requires time, expensive equipment, and trained staff. The pumps are noisy and need recharging and calibration. Dust is sampled using several types of filters and sampling heads constructed for collection of particles with defined size (e.g., inhalable, respirable dust). Air may be sampled stationary by low (2–20 L/min) or high volume (60–1100 L/min) pumps, or using person-carried pumps often at flow rates of 2 or 3.5 L/min. Different sample volumes and collection times directly affect the lower detection limits, and dust amounts are strongly dependent on activities in the room causing air disturbance. Results are expressed as allergen per cubic meter of air. In comparison to reservoir dust, airborne dust may be considered a more representative measure of inhaled allergen. Personal sampling in the breathing zone used for task and shift measurements is regarded as a gold standard in occupational settings.

Another method that can be used to collect airborne dust is the ion-charging device (28). This technique is based on a commercial air cleaner, where particles in the air are loaded with a positive charge, which allows them to attract and bind to the negatively charged collector plates. In general, airborne measurements are rarely performed in home environments because of high logistic costs.

*Settling or passive airborne dust* sampling is an alternative or a complement to the other two sampling techniques. This method collects airborne dust that has settled over a period of time (e.g., 2 weeks) on a certain sampling height (e.g., 1.5 m above the floor). In recent years, several sampling devices have been used such as Petri dishes (29), aluminum foil-covered boxes (30), or electrostatic cloths (31). Results are expressed in allergen per square meter (and per day). Settling dust seems to correlate moderately with both airborne dust as well as reservoir dust. Due to its low cost, ease of use, and simple transport, this method is suitable for large-scale exposure studies.

### Allergen analysis

Standard methods for allergen quantification are immunoassays based on specific antibodies directed against the allergens. A broad panel of assays has been developed (32), but some methods such as quantitative immunelectrophoresis or ELISA inhibition are hardly used. In the past two decades, ELISAs (enzyme linked immunosorbent assay) in “sandwich” design have been established as the gold standard for allergen analysis (33). The antibodies may be either monoclonal or polyclonal against either allergen mixtures or single allergens. Variations occur according to the source, specificity and purity of antibodies. In particular, the choice of standard (e.g., animal dander extract or purified or recombinant major allergens) and its protein determination method (Bradford-, BCA-, Lowry-assay, amino acid analysis) may influence the resulting values up to several orders of magnitude. Furthermore, several detection (conjugates) and visualization (substrates) methods are available.

The increasing interest and need to quantify allergens in different environments in recent years has led to the development of multiplex arrays for indoor allergens (MARIA) (34). Multiplex technology uses the same (or equivalent) antibody combinations used in ELISAs. Capture antibodies are covalently coupled to polystyrene beads that are internally labeled with fluorophores. Combining different bead types with different antibodies allows simultaneous measurement of several allergens in a single test.

## Cats and Dogs

The popularity of cats and dogs as pets means that allergy to both animals affects the general population. In Europe, the frequency of pet ownership is highly variable. Cat ownership ranged from 7.2 to 35% (average 14.9%) and dog ownership from 5.4 to 35% (average 12%) across 12 European birth cohorts with a total of 25,056 subjects (35). In the United States, according to the American Pet Products Manufacturers Association, nearly 40 and 33% of households own dogs and cats, respectively (36). Equally, the prevalence of sensitization also varies in different countries because of cultural differences and environmental factors. A large patient-based study GA^2^LEN (The Global Asthma and Allergy European Network) investigated the sensitization patterns for diverse inhalant indoor and outdoor allergens across 14 European countries using skin prick testing (5). The overall European sensitization frequency to cats and dogs were very similar, but regional differences were found. Cat sensitization rate was 26.3%, ranging from 16.8 to 49.3%. The rate of sensitization to dog allergens was 27.2%, ranging from 16.1 to 56%. Sensitization rates to both allergens were particularly high in Nordic countries (e.g., Denmark and Finland) and lower in Central/Western and Mediterranean countries (e.g., Austria, Belgium, Italy). In the US Inner City Asthma Study, skin test sensitivity among asthmatic children was 41% to cat allergens and 21% to dog allergens (37). Naturally, in the general population, the sensitization frequencies are much lower compared with those detected in patient populations (5). In a recent survey, performed in Germany (38), 7% of 7025 adult participants were sensitized to cat as well as to dog allergens (detection of specific IgE). Allergy to pets may also occur in some professions where workers are heavily exposed to animal dander during most of their working time. Work-related allergic symptoms have been reported in animal laboratory workers (30% to cats, 25% to dogs) and veterinarians (26% to cats, 19% to dogs) when handling animals (39, 40). However, it has to be considered that these persons may also handle animals before and/or outside of their respective careers.

### Cat (*Felis domesticus*)

Cat dander contains several allergens. The major cat allergen Fel d 1 is the most extensively studied animal allergen with regard to its structure, aerodynamic properties, environmental distribution, and the relationship between allergen exposure and the development of allergic disease (41, 42). Fel d 1 is a tetrameric glycoprotein formed by two heterodimers and has an apparent molecular weight of about 38 kDa (43). It reacts with IgE from over 90% of cat-sensitized individuals (44). In contrast to other animal major allergens, Fel d 1 is not a lipocalin. The three dimensional structure of Fel d 1 is very similar to that of uteroglobins, anti-inflammatory proteins, but its biological function is still unknown (45). Fel d 1 is primarily found in cat skin and hair follicles and is produced in sebaceous, anal, and salivary glands (46–48). It is transferred to the fur by licking and grooming. Male cats produce a larger amount of Fel d 1 than female cats (49). Fel d 1 was mainly associated with particles >9 μm, representing approximately 49% of the total allergen recovered. About 23% of airborne Fel d 1 was carried on small particles <4.7 μm (12). Other cat allergens are: Fel d 2 (67 kDa), serum albumin; Fel d 3 (11 kDa), cystatin; the second major cat allergen Fel d 4 (20 kDa), lipocalin; Fel d 5, immunoglobulin A; Fel d 6, immunoglobulin M; Fel d 7 (18 kDa), von Ebner gland protein; and Fel d 8 (24 kDa), latherin (11, 50).

### Dog (*Canis familiaris*)

Hair/dander and saliva are the main sources of dog allergens. The major dog allergen, Can f 1 (about 22–24 kDa) belongs to the lipocalin family of proteins and is produced in tongue epithelial tissue (51). About, 70% of dog allergic subjects have been shown to have IgE directed to Can f 1 (51, 52). Similar to Fel d 1 in cats, males produce more Can f 1 than females (53). The particle size distribution of Can f 1 is similar to that of Fel d 1 as well (54). Although, differences in Can f 1 allergen production have been found between different dog breeds, the variability between individuals is very large and a hypoallergenic breed does not exist (53, 55). Further dog allergens are: Can f 2 (24–27 kDa), lipocalin; Can f 3 (67 kDa), serum albumin; Can f 4 (16 kDa), lipocalin; Can f 5 (28 kDa), prostatic kallikrein; and Can f 6 (27–29 kDa), lipocalin (11, 50).

### Pet exposures

There are an overwhelming number of publications concerning the quantitative measurements of cat and dog allergens. Studies have been conducted worldwide but the research has been most active in the United States and in European countries. Study results are presented in Table 1 for cat allergens and in Table 2 for dog allergens. Most of the investigations chosen have estimated the levels of both cat and dog allergens in parallel. A further common feature of these studies is the usage of the same (or very similar) quantification method. The sandwich ELISA for cat allergens is based on two monoclonal antibodies, 6F9 and 3E4 against Fel d 1 (56). The sandwich ELISA for dog allergens is a combination of a monoclonal capture antibody 6E9 and polyclonal detection antibody against Can f 1 (57). Both assays are commercially available by Indoor Biotechnologies. Currently, Fel d 1 and Can f 1 can also be quantified with the multiplex array MARIA (Indoor Biotechnologies). In this system, the original Can f 1 ELISA was modified to use two monoclonal antibodies, 10D4 for capture and biotinylated 6E9 for detection. Commercial Fel d 1 and Can f 1 assays use purified natural single allergens as the standard quantified by amino acid analysis, but variations can occur in the protein standard used, which is not always specified in the relevant publications.

**Table 1:** **Studies related to exposure assessment to cat allergen**.

Study	Environment/country	Assay	Sampling method	Allergen level	
Custovic et al. (58)	Homes, UK	Fel d 1	Air samples (high volume pumps)		Range
		Sandwich ELISA	Homes with cats, *n* = 34		0.4–22.3 ng/m^3^
		(mAB)	Homes without cats, *n* = 62		<LOD–1.8 ng/m^3^
			Reservoir dust from living room floor	GM	95% CI
			Homes with cats, *n* = 34	204.5 μg/g	108.5–385.3 μg/g
			Homes without cats, *n* = 62	0.8 μg/g	0.61–1.06 μg/g
Custis et al. (59)	Homes, USA	Fel d 1	Air samples (ion-charging device)	GM	
		Sandwich ELISA	Homes with cats		
		(mAB)	Living room, *n* = 27	1.52 μg/day	
			Bedroom, *n* = 16	1.12 μg/day	
			Homes without cats		
			Living room, *n* = 17	0.049 μg/day	
			Bedroom, *n* = 13	0.042 μg/day	
Arbes et al. (60)	Homes, USA	Fel d 1	Reservoir dust	GM	
		Sandwich ELISA	House index^a^, *n* = 825	4.73 μg/g	
		(mAB)	Bedroom bed, *n* = 728	2.74 μg/g	
			Bedroom floor, *n* = 736	2.13 μg/g	
			Living room floor, *n* = 759	2.14 μg/g	
			Living room sofa, *n* = 717	6.17 μg/g	
			Homes with cats, *n* = 187	199.7 μg/g	
			Homes without cats, *n* = 630	1.47 μg/g	
Heinrich et al. (61)	Homes, across Europe (several countries)	Fel d 1	Reservoir dust from mattresses	GM	95% Range^b^
		Sandwich ELISA	Total, *n* = 2800	0.94 μg/g	0.84–1.05 μg/g
		(mAB)	Central Europe *n* = 1023	1.79 μg/g	1.48–2.15 μg/g
			Northern Europe, *n* = 767	1.45 μg/g	1.17–1.79 μg/g
			Southern Europe, *n* = 1010	0.35 μg/g	0.30–0.41 μg/g
			Never cat owners, *n* = 2044	0.29 μg/g	0.01–8.3 μg/g
			Past cat owners, *n* = 192	1.37 μg/g	0.01–149.3 μg/g
			Current cat owners, *n* = 555	61.40 μg/g	0.22–17,072 μg/g
Simons et al. (62)	Homes, USA	Fel d 1	Reservoir dust from bedroom	GM	
		Sandwich ELISA	Inner city, *n* = 98	0.75 μg/g	
		(mAB)	Suburban, *n* = 19	2.4 μg/g	
			Homes with cats	16.9 μg/g	
			Homes without cats	0.43 μg/g	
Bertelsen et al. (63)	Homes, Norway	Fel d 1	Reservoir dust from mattresses	GM	95% CI
		Sandwich ELISA	All homes, *n* = 797	1.32 μg/g	1.14–1.54 μg/g
		(mAB)	Girls, *n* = 360	1.93 μg/g	1.50–2.47 μg/g
			Boys, *n* = 437	0.97 μg/g	0.81–1.17 μg/g
			Homes without cats, *n* = 640	0.62 μg/g	0.54–0.86 μg/g
			Girls, *n* = 276	0.74 μg/g	0.63–0.86 μg/g
			Boys, *n* = 364	0.55 μg/g	0.48–0.62 μg/g
Wardzynska et al. (75)	Homes, Poland	Fel d 1	Reservoir dust from mattresses	Median	IQR
		Sandwich ELISA	Homes with cats	
		(mAB)	Rural, *n* = 23	0.62 μg/g	0.42–0.77 μg/g
			Urban, *n* = 12	0.80 μg/g	0.65–1.07 μg/g
			Homes without cats	
			Rural, *n* = 168	0.42 μg/g	0.26–0.61 μg/g
			Urban, *n* = 129	0.59 μg/g	0.26–0.83 μg/g
Perzanowski et al. (64)	Schools and homes, Sweden	Fel d 1	Reservoir dust	GM	Range
		Sandwich ELISA	Schools, *n* = 176	0.76 μg/g	<LOD–13 μg/g
		(mAB)	Desk and chairs, *n* = 64	2.6 μg/g	<LOD–13 μg/g
			Floors, *n* = 64	0.31 μg/g	<LOD–8.8 μg/g
			Homes without cats, *n* = 74	0.42 μg/g	<LOD–9.4 μg/g
			Homes with cats, *n* = 9	33 μg/g	1.8–950 μg/g
Karlsson et al. (29)	Schools, Sweden	Fel d 1	Petri dishes (5 days)	Median	
		Sandwich ELISA	Many cat owners in class, *n* = 22	81.0 ng/m^2^/day	
		(mAB)	Few cat owners in class, *n* = 22	14.2 ng/m^2^/day	
			Air personal samples (pumps)		
			Many cat owners in class, *n* = 22	1.6 ng/m^3^
			Few cat owners in class, *n* = 22	0.3 ng/m^3^
Arbes et al. (65)	Day-care centers, USA	Fel d 1	Reservoir dust from floors	GM	
		Sandwich ELISA	89 Day-care centers	1.43 μg/g	
		(mAB)	20 Day-care centers		
			Carpet	2.28 μg/g	
			Smooth floor	0.39 μg/g	
Tranter et al. (66)	Schools and day-care centers, USA	Fel d 1	Reservoir dust from floors	Median	IQR
		Sandwich ELISA	Schools	
		(mAB)	Carpet, *n* = 79	0.28 μg/m^2^	0.14–0.77 μg/m^2^
			Tile, *n* = 65	0.014 μg/m^2^	0.006–0.027 μg/m^2^
			Day-care centers	
			Carpet, *n* = 42	0.42 μg/m^2^	0.14–0.9 μg/m^2^
			Furniture, *n* = 18	1.6 μg/m^2^	0.74–4.0 μg/m^2^
Cai et al. (67)	Day-care centers, Sweden	Fel d 1	Petri dishes (30–40 days)	GM	Range
		Sandwich ELISA	Diverse rooms, *n* = 97	9.4 ng/m^2^/day	0.9–78.6 ng/m^2^/day
		(mAB)			
Permaul et al. (68)	Schools and homes, USA	Fel d 1	Reservoir dust	GM	Range
		MARIA	Schools, *n* = 229	0.19 μg/g	0.004–285.8 μg/g
		(mAB)	Homes (bedroom), *n* = 118	0.06 μg/g	0.004–392.3 μg/g
			Air samples (ion-charging device)		
			Schools, *n* = 196	1.82 ng/m^3^	
Custovic et al. (69)	Hospitals, UK	Fel d 1	Reservoir dust from upholstered	GM	Range
		Sandwich ELISA	chairs, *n* = 42	22.9 μg/g	4.5–58 μg/g
		(mAB)	Air samples (high volume pumps)		<LOD–0.22 ng/m^3^
Partti-Pellinen et al. (70)	Public transport vehicles, Finland	Fel d 1	Reservoir dust	Median	Range
		Sandwich ELISA	Seats, *n* = 8	0.87 μg/g	0.003–2.6 μg/g
		(mAB)	Floors, *n* = 10	0.01 μg/g	0.002–0.08 μg/g
Macher et al. (71)	Offices, USA	Fel d 1	Reservoir dust from floors	Median	Range
		Sandwich ELISA	92 offices, *n* = 251	0.3 μg/g	<LOD–19 μg/g
		(mAB)			
Samadi et al. (72)	Animal hospital, Netherlands	Fel d 1	Different locations	GM	Range
		Sandwich ELISA + MARIA(mAB)	Air personal samples (pumps), *n* = 67	0.3 ng/m^3^	<LOD–9.4 ng/m^3^
			Electrostatic cloths (14 days), *n* = 30	56 ng/m^2^	<LOD–579 ng/m^2^
			Reservoir dust from floors, *n* = 110	11 ng/m^2^	0.2–183 ng/m^2^

*mAB, monoclonal antibodies; GM, geometric mean; IQR, interquartile range; CI, confidence interval; LOD, limit of detection*.

*^a^House index represents the mean of the sample location concentrations*.

*^b^95% Range calculated as log mean plus and minus two standard deviations and back-transformed*.

**Table 2:** **Studies related to exposure assessment to dog allergen**.

Study	Environment/country	Assay	Sampling method	Allergen level	
Custovic et al. (58)	Homes, UK	Can f 1	Air samples (high volume pumps)		Range
		Sandwich ELISA	Homes with dogs, *n* = 31		0.5–99 ng/m^3^
		(mAB/pAB)	Homes without dogs, *n* = 62		<LOD–12.4 ng/m^3^
			Reservoir dust from living room floor	GM	95%CI
			Homes with dogs, *n* = 31	181.3 μg/g	102.0–322.3 μg/g
			Homes without dogs, *n* = 62	1.56 μg/g	1.17–2.08 μg/g
Custis et al. (59)	Homes, USA	Can f 1	Air samples (ion-charging device)	GM	
		Sandwich ELISA	Homes with dogs		
		(mAB/pAB)	Living room, *n* = 17	1.48 μg/day	
			Bedroom, *n* = 10	2.80 μg/day	
			Homes without dogs		
			Living room, *n* = 27	<LOD	
			Bedroom, *n* = 19	0.116 μg/day	
Arbes et al. (60)	Homes, USA	Can f 1	Reservoir dust	GM	
		Sandwich ELISA	House index^a^, *n* = 825	4.69 μg/g	
		(mAB/pAB)	Bedroom bed, *n* = 682	2.48 μg/g	
			Bedroom floor, *n* = 718	2.99 μg/g	
			Living room floor, *n* = 731	3.61 μg/g	
			Living room sofa, *n* = 690	5.49 μg/g	
			Homes with dogs, *n* = 247	69.23 μg/g	
			Homes without dogs, *n* = 570	1.33 μg/g	
Simons et al. (62)	Homes, USA	Can f 1	Reservoir dust from bedroom	GM	
		Sandwich ELISA	Inner city, *n* = 98	0.38 μg/g	
		(mAB/pAB)	Suburban, *n* = 19	5.5 μg/g	
			Homes with dogs	13.4 μg/g	
			Homes without dogs	0.17 μg/g	
Nicholas et al. (73)	Homes, USA	Can f 1	Reservoir dust from bedroom floor	GM	95% CI
		Sandwich ELISA	Homes with dogs, *n* = 254	1.24 μg/g	0.91–1.71 μg/g
		(mAB/pAB)	Dogs indoors, *n* = 219	1.59 μg/g	1.14–2.21 μg/g
			Dogs outdoors only, *n* = 30	0.13 μg/g	0.07–0.23 μg/g
			Homes without dogs, *n* = 738	0.055 μg/g	0.048–0.063 μg/g
Bertelsen et al. (63)	Homes, Norway	Can f 1	Reservoir dust from mattresses	GM	95% CI
		Sandwich ELISA	All homes, *n* = 797	0.61 μg/g	0.53–0.73 μg/g
		(mAB/pAB)	Girls, *n* = 360	0.78 μg/g	0.62–0.98 μg/g
			Boys, *n* = 437	0.50 μg/g	0.41–0.62 μg/g
			Homes without dogs, *n* = 674	0.31 μg/g	0.27–0.34μg/g
			Girls, *n* = 297	0.37 μg/g	0.31–0.44 μg/g
			Boys, *n* = 374	0.26 μg/g	0.23–0.30 μg/g
Wardzynska et al. (75)	Homes, Poland	Can f 1	Reservoir dust from mattresses	Median	IQR
		Sandwich ELISA	Homes with dogs
		(mAB/pAB)	Rural, *n* = 13	0.55 μg/g	0.17–2.47 μg/g
			Urban, *n* = 57	5.0 μg/g	3.25–7.07μg/g
			Homes without dogs
			Rural, *n* = 176	0.35 μg/g	0.22–0.64 μg/g
			Urban, *n* = 84	1.55 μg/g	0.85–2.44μg/g
Vredegoor et al. (55)	Homes, Netherlands	Can f 1	Electrostatic cloths (28 days)	GM	
		Sandwich ELISA	Homes of dog owners, *n* = 168		
		(mAB/pAB)	Labradoodle, *n* = 54	5.22 μg/m^2^	
			Labrador retriever, *n* = 25	5.04 μg/m^2^	
			Poodle, *n* = 23	6.32 μg/m^2^	
			Spanish waterdog, *n* = 13	9.05 μg/m^2^	
			Airedale terrier, *n* = 22	8.44 μg/m^2^	
			Rest, *n* = 31	7.18 μg/m^2^
Perzanowski et al. (64)	Schools and homes, Sweden	Can f 1	Reservoir dust	GM	Range
		Sandwich ELISA	Schools, *n* = 176	3.3μg/g	<LOD–176 μg/g
		(mAB/pAB)	Desk and chairs, *n* = 64	15 μg/g	0.45–176 μg/g
			Floors, *n* = 64	1.1 μg/g	<LOD–60 μg/g
			Homes without pets, *n* = 74	2.0 μg/g	<LOD–22 μg/g
			Homes with dogs, *n* = 29	79 μg/g	13–625 μg/g
Arbes et al. (65)	Day-care centers, USA	Can f 1	Reservoir dust from floors	GM	
		Sandwich ELISA	89 Day-care centers	2.06 μg/g	
		(mAB/pAB)	20 Day-care centers		
			Carpet	2.13 μg/g	
			Smooth floor	0.29 μg/g	
Tranter et al. (66)	Schools and day-care centers, USA	Can f 1	Reservoir dust	Median	IQR
		Sandwich ELISA	Schools	
		(mAB/pAB)	Carpet, *n* = 79	0.45 μg/m^2^	0.23–1.4 μg/m^2^
			Smooth floor, *n* = 65	0.03 μg/m^2^	0.014–0.053 μg/m^2^
			Day-care centers		
			Carpet, *n* = 42	0.44 μg/m^2^	0.18–0.96 μg/m^2^
			Furniture, *n* = 18	1.1 μg/m^2^	0.40–2.2 μg/m^2^
Cai et al. (67)	Day-care centers, Sweden	Can f 1	Petri dishes (30–40 days)	GM	Range
		Sandwich ELISA	Diverse rooms, *n* = 97	7.2 ng/m^2^/day	1.2–72.5 ng/m^2^/day
		(mAB/pAB)			
Permaul et al. (68)	Schools and homes, USA	Can f 1	Reservoir dust	GM	Range
		MARIA	Schools, *n* = 229	0.08 μg/g	0.004–285.8 μg/g
		(mAB)	Homes (bedroom), *n* = 118	0.03 μg/g	0.004–392.3 μg/g
			Air samples (ion-charging device)		
			Schools, *n* = 196	1.17 ng/m^3^	
Custovic et al. (74)	Public places, UK	Can f 1	Reservoir dust		Range
		Sandwich ELISA	Total (5 schools, 6 hotels, 4 cinemas, 6 pubs, 3 buses, 2 trains)		0.2–52.5 μg/g
		(mAB/pAB)		GM	95% CI
			Upholstered seats	9.4 μg/g	6.4–13.9 μg/g
			Carpets	1.5 μg/g	1.3–1.7 μg/g
Custovic et al. (69)	Hospitals, UK	Can f 1	Reservoir dust from upholstered	GM	Range
		Sandwich ELISA	chairs, *n* = 42	21.6 μg/g	4.0–63 μg/g
		(mAB/pAB)	Air stationary samples (pumps), *n* = 10		0.12–0.56 ng/m^3^
Partti-Pellinen et al. (70)	Public transport vehicles, Finland	Can f 1	Reservoir dust	Median	Range
		Sandwich ELISA	Seats, *n* = 8	2.4 μg/g	0.02–8.5 μg/g
		(mAB/pAB)	Floors, *n* = 10	0.2 μg/g	0.004–0.86 μg/g
Samadi et al. (72)	Animal hospital, Netherlands	Can f 1	Different locations	GM	Range
		Sandwich ELISA + MARIA(mAB)	Air personal samples (pumps), *n* = 67	3.6 ng/m^3^	<LOD–73.3 ng/m^3^
			Electrostatic cloths (14 days), *n* = 30	720 ng/m^2^	<LOD–12,105 ng/m^2^
			Reservoir dust from floors, *n* = 110	7.1 ng/m^2^	<LOD–13,644 ng/m^2^

*mAB, monoclonal antibodies; pAB, polyclonal antibodies; GM, geometric mean; IQR, interquartile range; CI, confidence interval; LOD, limit of detection*.

*^a^House index represents the mean of the sample location concentrations*.

The most important finding of all studies is that cat and dog allergens are ubiquitously found in every type of human indoor environment, regardless of the presence of pets, most likely due to passive transfer via clothing. Exposure levels vary widely between different environments and geographical regions. For example, a multicenter cross-sectional study measured Fel d 1 levels in mattress dust from 2800 households in 22 municipal areas across Europe (61). European regions showed substantial differences with respect to allergen levels, with the highest concentrations of Fel d 1 found in central European countries followed by the northern and finally southern countries. The overall geometric mean was 0.94 μg/g, ranging from the lowest measured value of 0.12 μg/g in Huelva, Spain to the highest of 3.76 μg/g in Antwerp, Belgium. The major strength of this study was the good comparability among different regions because the identical dust collection protocol was used, and Fel d 1 measurements were performed in one single laboratory using identical batches of ELISA kits.

Apart from the high variability, exposure intensity/degree is primarily related to the presence (past or present) of a pet in the home. Not surprisingly, cat and dog allergen concentrations were found to be much higher in homes with pets than in homes without pets, as shown using both reservoir dust samples (58, 60–62, 64, 73) and air samples (58, 59). In settled dust, there was an 80- to 250-fold difference for Fel d 1, and 25- to 120-fold difference for Can f 1 between houses with and without pets. Higher geometric mean of cat allergens has been also estimated in homes where cats were once present compared with those that never housed cats (61). Homes with outdoor dogs had significantly higher dog allergen levels than homes without any dogs, but significantly lower levels than homes with indoor dogs (73). Another multicenter cross-sectional survey from the United States investigated the distribution of Can f 1 and Fel d 1 within households according to the sampling site and vacuumed surface (60). Independent of the presence of pets, the highest concentrations of both allergens were found in living room sofas. In homes with pets, such high concentrations generally indicated the favorite indoor location of the animals; whereas, in homes without pets, such sites were those that came into most contact with clothing. Additionally, sofas are generally less frequently cleaned than floors or bedding.

Different infrastructural characteristics (urban, suburban, rural) also appear to influence the allergen exposure intensity. Suburban homes contained higher levels of cat and dog allergens than inner city homes, probably reflecting the higher rate of pet ownership in these households (62). In rural homes, the median concentrations of cat and dog allergens in mattresses were significantly lower than in those from urban houses, probably reflecting the different habits of pet owners (75). In rural areas, animals are usually kept outdoors, whereas more indoors pets are found in cities. Moreover, a study from Norway has reported that gender-specific differences can occur in pet allergen exposure among children (63). Girls had higher concentrations of cat and dog allergens in their mattresses compared with boys, also after adjustment for pet ownership. This was most likely caused by differences in behavior, such as a greater affinity among girls to cuddle pets or the tendency to decorate their rooms with soft toys, which may act as reservoirs for allergens.

To explore the differences in allergen distribution among different dog breeds, allergen levels in settled airborne dust (electrostatic cloths) were measured in homes of various so-called hypoallergenic (Labradoodle, Poodle, Spanish Waterdog, Airedale terrier) and non-hypoallergenic dogs (Labrador retriever, control heterogeneous group). Despite Can f 1 differences in hair samples between hypoallergenic and non-hypoallergenic dogs (with enormous variability between individual dogs of the same breed), no differences were observed in environmental levels. Surprisingly, airborne Can f 1 concentrations in homes were similar across different breeds and no evidence was found for a reduced production of allergen by hypoallergenic dogs (55).

Besides domestic settings, many studies on indoor allergens have focused on schools and day-care centers due to lengthy periods that children spend in these locations. A comparison of settled dust samples between these two types of environments suggests that cat and dog allergen levels in schools are higher than in homes where no pets are present (64, 68). These results clearly demonstrate that educational facilities may be the most important site of persistent exposure for some children and thus a risk factor, especially for those who are allergic or asthmatic. Higher allergen concentrations were found in furniture compared to floors (64, 66), consistent with what was described above for personal home environments. Furthermore, carpeted floors contained higher allergen levels than smooth floors (65, 66). Fel d 1 and Can f 1 were also detectable in active airborne dust (ion-charging device) from schools (68) and passive airborne dust (Petri dishes) from day-care centers (67). Karlsson et al. (29) have shown that airborne Fel d 1 concentrations in schools were strongly dependent on the number of children with cats at home. Median cat allergen levels in both Petri dishes and personal air samples were approximately fivefold higher in classes with many cat owners (>20%) compared to classes with few cat owners (<10%).

The widespread distribution of cat and dog allergens is further demonstrated by several studies carried out in other public indoor environments. Fel d 1 and Can f 1 have been measured in reservoir dust from seats and floors inside public buildings such as hotels, cinemas and pubs (74), hospitals (69), office buildings (71), and in public transport vehicles such as buses, trams, and trains (70, 74). In general, seats were more contaminated with pet allergens compared to floors, presumably because they come in direct contact with clothing.

Reports on measuring exposure to pet allergens in occupational settings are rare. One study investigated the allergen levels in veterinary medicine students, and workers in a companion animal hospital using various dust collection methods throughout different locations (72). In personal airborne samples, significant differences in exposure levels were found for Fel d 1 and Can f 1 between the different performed tasks. The highest exposure was observed in the intensive care unit and during practical animal courses for students. Allergen concentrations varied greatly between different locations, with the highest levels measured in the examination and waiting rooms (reservoir dust) and in the intensive care unit (electrostatic cloths) and the lowest in the operating room (both methods). Exposure levels in dust captured by diverse sampling methods only correlated moderately with each other.

## Rodents

Allergy to mice and rats is an important occupational health problem, primarily because these animals are the most widely used in medical research. Rodent allergy is commonly observed among technicians, animal caretakers, physicians, and scientists who work in pharmaceutical industries, university laboratories, and animal breeding facilities. In occupational settings, the prevalence rates of rodent allergies vary from 11 to 44% depending on the diagnostic methods (questionnaire or laboratory testing) used (76). The prevalence of mouse and rat allergy is very similar. In a large cross-sectional survey from Japan that included over 5000 laboratory animal workers, 26% reported allergic symptoms to mice and 25% to rats (39). Sensitization and work-related symptoms occur at the latest, 2–3 years after the initial exposure to laboratory animals (77). Besides an atopic background, the most important risk factor for the development of an allergy to rodents is the level of exposure to laboratory animal allergens. Children of parents who are occupationally exposed to laboratory animals were shown to have more frequently positive skin prick tests against mice, rats, and hamsters compared to children of non-exposed parents (78). Studies from the United States have demonstrated that rodent allergen exposure in domestic environments is also clinically relevant. In inner city children with asthma, the prevalence of mouse sensitization (skin test sensitivity) ranged from 11 to 46% (79), and the prevalence of rat sensitization was estimated to be 21% (80). A recent study from Europe has reported a very low sensitization prevalence (1.6% for mouse and 0.6% for rat) in urban atopic populations without occupational exposure (81).

### Mouse (*Mus musculus*)

Rodent allergens can be found in dander, hair, urine, saliva, and serum. Urine is the main source of allergenic proteins in both mice and rats. The major mouse allergen, Mus m 1 (about 19 kDa) is a prealbumin and lipocalin–odorant binding protein (82) belonging to the rodent family of major urinary proteins (MUP) (83). MUPs are produced in the liver and other exocrine glands under hormonal control. Mouse MUPs are encoded by 35 genes, with 15 forms detectable in urine. The expression of MUPs varies according to species, strains, sexes, and individuals. MUPs seem to play a complex role in chemosensory signaling among rodents. Study of particle size distribution revealed that airborne Mus m 1 is carried on particles with aerodynamic diameter ranging from 0.4 to >10 μm, with the majority on particles between 3.3 and 10 μm (84).

To quantify mouse allergens, sandwich ELISAs were developed by several groups using polyclonal antibodies produced in rabbit or sheep (84–86). In these studies, either the purified Mus m 1 or the whole protein from mouse urine was used as the immunogen or standard. Therefore, some assays are known in the literature as mouse urinary allergen (MUA) or MUP. A polyclonal antibody-based assay is commercially available as the Mus m 1 ELISA kit or as a component of the MARIA (Indoor Biotechnologies).

Numerous studies of mouse allergen exposure levels were conducted in occupational settings such as laboratory animal facilities of universities, research institutes, and pharmaceutical companies (Table 3). Ohman et al. (84) demonstrated that Mus m 1 is widely distributed in the air of a major mouse breeding facility, even in rooms that do not house mice (e.g., offices). This was shown for stationary as well as for personal air samples. Direct contact to mice was associated with the highest Mus m 1 levels (up to 560 ng/m^3^). Within mouse rooms, airborne Mus m 1 levels were strongly correlated with the number of mice in the room. Another large-scale study (seven different facilities) investigated the personal allergen exposure intensity according to the type of job and the type of tasks performed (85). Animal technicians and caretakers had elevated median MUA exposure levels compared with the scientific staff and supervisors. Removal of contaminated bedding from cages and moving animals into new cages were associated with the highest personal exposure; lower exposures were seen with feeding or handling the animals. However, mouse allergen concentrations during tasks varied enormously among different facilities (<LOD–2000 ng/m^3^), probably due to the differences in cleaning practices and feeding technologies, or the local ventilation equipment.

**Table 3:** **Studies related to exposure assessment to mouse allergen**.

Study	Environment/country	Assay	Sampling method	Allergen level	
Ohman et al. (84)	Mouse facility, USA	Mus m 1 Sandwich ELISA (pAB)	Air samples		
			Stationary	Range of AM	
			Mouse rooms	0.5–15.1 ng/m^3^	
			Offices and lunch room	0.2–1.5 ng/m^3^	
			Personal		
			Mouse rooms	16.6–563 ng/m^3^	
			Offices and lunch room	1.2–2.7 ng/m^3^	
Hollander et al. (85)	Laboratory animal facilities, Netherlands	MUA	Air samples (personal)		
		Sandwich ELISA (pAB)	Job categories, *n* = 171	Median	
			Animal caretaker, *n* = 63	12.1 ng/m^3^	
			Animal technicians, *n* = 94	6.4 ng/m^3^	
			Scientists, *n* = 2	2.7 ng/m^3^	
			Supervisor, *n* = 12	0.58 ng/m^3^	
			Task categories, *n* = 123	GM	Range
			Cage emptying, *n* = 25	74.8 ng/m^3^	<LOD–2700 ng/m^3^
			Changing animals, *n* = 33	22.8 ng/m^3^	<LOD–501 ng/m^3^
			Feeding animals, *n* = 19	19.6 ng/m^3^	<LOD–542 ng/m^3^
			Handling animals, *n* = 8	16.0 ng/m^3^	<LOD–209 ng/m^3^
			Experiments, *n* = 2	33.5 ng/m^3^	8–140 ng/m^3^
			Biotechnical work, *n* = 11	5.4 ng/m^3^	<LOD–51 ng/m^3^
			Cage wash, *n* = 8	2.6 ng/m^3^	<LOD–89 ng/m^3^
			Cleaning rooms, *n* = 17	2.1 ng/m^3^	<LOD–151 ng/m^3^
Curtin-Brosnan et al. (87)	Mouse facility, USA	Mus m 1 Sandwich ELISA (pAB)	Air samples (personal)	Median	IQR
			Mouse handlers, *n* = 97	4.13 ng/m^3^	0.70–12.12 ng/m^3^
			Non-mouse handlers, *n* = 71	0.21 ng/m^3^	<LOD–0.63 ng/m^3^
			Job categories		Range
			Animal caretaker, *n* = 57	9.6 ng/m^3^	0.58–220.9 ng/m^3^
			Administrative personnel, *n* = 34	0.23 ng/m^3^	<LOD–30.94 ng/m^3^
			Supplies/Material handler, *n* = 19	0.63 ng/m^3^	<LOD–423.9 ng/m^3^
			Task categories (mouse handlers)		IQR
			Animal care, *n* = 42	8.73 ng/m^3^	3.56–18.68 ng/m^3^
			Husbandry, *n* = 26	5.83 ng/m^3^	3.26–14.95 ng/m^3^
			Experiments, *n* = 25	0.36 ng/m^3^	0.07–1.77 ng/m^3^
Renström et al. (86)	Laboratory animal facility, Sweden	MUA	Air samples (stationary)	Median	IQR
		Sandwich ELISA (pAB)	Open cages, *n* = 11	44 ng/m^3^	36–45 ng/m^3^
			IVC 1, *n* = 13	0.62 ng/m^3^	<LOD–2.4 ng/m^3^
			IVC 2, *n* = 15	4.3 ng/m^3^	2.7–8.8 ng/m^3^
Thulin et al. (88)	Laboratory animal facility, Sweden	MUA	Air samples (personal)		
		Sandwich ELISA	Cage changing	GM	Range
		(pAB)	Unventilated table, *n* = 5	77.3 ng/m^3^	65.1–88 ng/m^3^
			Ventilated changing wagon, *n* = 5	17.2 ng/m^3^	14.0–20.8 ng/m^3^
			Handling animals		
			Outside ventilated bench, *n* = 9	87.2 ng/m^3^	34.8–220 ng/m^3^
			Ventilated bench, *n* = 6	2.1 ng/m^3^	0.6–9.8 ng/m^3^
Krop et al. (25)	Homes of animal caretakers, Netherlands	MUA	Reservoir dust from mattresses	GM	95% CI
		Sandwich ELISA	Laboratory animal workers, *n* = 15	29.5 ng/g	11.7–74.6 ng/g
		(pAB)	Controls (no contact to animals), *n* = 15	8.8 ng/g	4.6–16.8 ng/g
Phipatanakul et al. (89)	Homes, USA	Mus m 1 Sandwich ELISA (pAB)	Reservoir dust from bed, furniture, floor	Median	Range
			Bedroom, *n* = 506	0.52 μg/g	<LOD–294 μg/g
			Living room, *n* = 608	0.57 μg/g	<LOD–203 μg/g
			Kitchen, *n* = 559	1.60 μg/g	<LOD–618 μg/g
Matsui et al. (90)	Homes, USA	Mus m 1 Sandwich ELISA (pAB)	Reservoir dust from bed, furniture, floor		
			Inner city	Median	IQR
			Bedroom, *n* = 78	0.76 μg/g	0.16–3.21 μg/g
			Living room, *n* = 77	0.99 μg/g	0.18–5.59 μg/g
			Kitchen, *n* = 75	2.48 μg/g	0.27–18.95 μg/g
			Suburban	
			Bedroom, *n* = 257	0.012 μg/g	<LOD–0.048 μg/g
			Living room, *n* = 250	0.016 μg/g	<LOD–0.044 μg/g
			Kitchen, *n* = 250	0.007 μg/g	<LOD–0.050 μg/g
Simons et al. (62)	Homes, USA	Mus m 1 Sandwich ELISA (pAB)	Reservoir dust from beds and floors		
			Bedroom	GM	
			Inner city, *n* = 98	3.2 μg/g	
			Suburban, *n* = 19	0.013 μg/g	
			Air samples		
			Bedroom		
			Inner city, *n* = 98	0.055 ng/m^3^	
			Suburban, *n* = 19	0.016 ng/m^3^	
Chew et al. (91)	Schools, USA	MUP	Reservoir dust from floors	Range of GM	Range of samples
		Sandwich ELISA	11 Schools, 87 classrooms	0.21–133 μg/g	<LOD–1125 μg/g
		(pAB)			
Arbes et al. (65)	Day-care centers, USA	Mus m 1	Reservoir dust from floors	GM	
		Sandwich ELISA	89 Day-care centers	0.01 μg/g	
		(pAB)	20 Day-care centers		
			Carpet	0.008 μg/g	
			Smooth floor	0.004 μg/g	
Sheehan et al. (92)	Schools and homes, USA	MUP	Reservoir dust	GM	Range
		Sandwich ELISA	Schools (floor, desks, chairs), *n* = 46	1.66 μg/g	<LOD–238 μg/g
		(pAB)	Homes (bedroom), *n* = 38	0.41 μg/g	<LOD–6.97 μg/g
Permaul et al. (68)	Schools and homes, USA	Mus m 1	Reservoir dust	GM	Range
		MARIA	Schools (floor, desks, chairs), *n* = 229	0.65 μg/g	0.001–544.4 μg/g
		(pAB)	Homes (bedroom), *n* = 118	0.10 μg/g	0.002–82.6 μg/g
			Air samples		
			Schools, *n* = 196	1.80 ng/m^3^	

*MUA, mouse urinary allergens; pAB, polyclonal antibodies; AM, arithmetic mean; GM, geometric mean; IQR, interquartile range; CI, confidence interval; LOD, limit of detection*.

A recent study from the Jackson Laboratory assessed mouse allergen exposure across a range of jobs, including non-mouse handlers (87). Although mouse handlers had significantly higher median levels of mouse allergen than non-handlers (4.13 vs. 0.21 ng/m^3^), 71% of administrative/support personnel and 68% of materials/supplies handlers had detectable mouse allergen levels, in some cases in concentrations similar to those measured in animal caretakers. Among mouse handlers, those involved in animal care or husbandry had higher allergen levels than those conducting laboratory experiments. Several studies examined the effects of caging systems and various ventilation and automation measures in reducing allergen levels. For example, in undisturbed animal rooms mouse allergen levels were much lower using individually ventilated cage (IVC) system compared to open cages (86). Using a ventilated cage changing wagon or handling animals on ventilated benches also resulted in lower exposure levels (88). Krop et al. (25) investigated the spreading of laboratory animal allergens outside the animal facilities. The authors found that levels of rodent allergens were significantly higher in mattress dust of laboratory animal workers compared with those of unexposed controls. In addition, high amounts of mouse allergens were recovered from hair-covering cups (not routinely used by laboratory animal workers), and therefore the authors concluded that the transfer of allergens via uncovered hair was the most likely cause for the spread of these allergens to the home.

In the past decade, the importance of rodent allergens outside of the workplace has been demonstrated in several studies (Table 3). Mouse allergen seems to be widespread in US communities and may be regarded as an environmental allergen. Phipatanakul et al. (89) analyzed house dust samples (bedroom, living room, and kitchen) from 608 homes of major inner city areas (New York, Baltimore, Chicago, Cleveland, Detroit, St Louis, Washington). Ninety-five percent of all homes had detectable Mus m 1 in at least one room, with the highest levels found in kitchens. Matsui et al. (90) compared the distribution of mouse allergen between inner city and suburban homes (surroundings of Baltimore). The prevalence of Mus m 1 was lower in suburban homes (e.g., bedrooms: 69 vs. 94%) and at approximately 100-fold lower concentrations (e.g., living room: 0.99 vs. 0.016 μg/g), compared with homes in the inner city. These differences were confirmed by another study based in Baltimore that analyzed both air samples and settled dust from bedrooms (62). The inner city homes also had significantly higher airborne mouse allergen levels than suburban homes, reflecting the higher rate of reported mouse infestation (80 vs. 5%) in cities.

Quantification of mouse allergen was also performed in school and day-care settings. Mouse allergen was detectable in 81% of settled dust samples collected from 11 schools in a major metropolitan area in the northeastern USA (91). Mouse allergen levels varied greatly between schools with geometric means (GM) ranging from 0.21 to 133 μg/g. In contrast, very low Mus m 1 (GM: 0.01 μg/g) concentrations were found in 86 day-care facilities in North Carolina, with a prevalence of 83% of all samples collected (65). The aim of two other studies was to investigate allergen exposure in schools compared with homes (68, 92). Both studies reported significantly higher levels of mouse allergen in classrooms vs. students’ bedrooms (settled dust samples). Mus m 1 was also detectable in airborne samples from schools using a very sensitive MARIA multiplex array. Airborne and settled dust Mus m 1 levels in classrooms were moderately correlated (*r* = 0.48; *p* < 0.0001).

### Rat (*Rattus norvegicus*)

Analogous to mouse allergens, the major rat allergen Rat n 1 (about 17 kDa) is a prealbumin or α-2u-globulin that belongs to the lipocalin group and to the family of MUPs. The amino acid identity between mouse and rat MUPs is approximately 65% (83, 93). A major difference between species is that MUPs are glycosylated in rats but not in mice. Furthermore, the urine of male rats contains much larger quantities of Rat n 1 than urine collected from female rats. Airborne rat allergen was detected on particles raging from >0.5 to 20 μm with the majority on particles larger than 8 μm (94).

The ELISA assays for rat allergen measurements are based on polyclonal antibodies against whole protein isolated from rat urine (85), or monoclonal antibodies against the major allergen Rat n 1 (80, 95). One assay based on the monoclonal antibodies RUP1 and RUP6 is commercially available by Indoor Biotechnologies as the Rat n 1 ELISA kit or as a component of the MARIA.

As for mice, occupational exposure to rat allergens is well documented in the literature, but with fewer published studies. For many studies, exposure to rodent allergens in the workplace was assessed in parallel, with the same findings (Table 4). Briefly, rat allergen levels in laboratory animal facilities are dependent on room, job, and task (85, 96). More specifically, airborne Rat n 1 was significantly higher in rat rooms than in experimental rooms, and the highest personal exposure was measured for animal technicians and caretakers, compared with students and scientists. Cage cleaning resulted in much higher rat allergen levels than animal handling. In addition, exposure varied strongly with facility. One study assessed the individual exposure to rodent allergens using nasal air samplers and demonstrated the effectiveness of personal respiratory protection equipment (97). This unique sampling device is worn inside the nostrils and controlled by the wearer’s breathing. The inhaled particles are collected by impaction on adhesive tape within the samplers. Using this method, clear differences in allergen levels (2.6 vs. 0.1 ng/h) were seen between high exposure tasks (manual cage emptying, animal handling) and low exposure tasks (automated cage changing, supervision). In addition, nasal air sampling correlated well with conventional sampling using air pumps (*r* = 0.8). The study also clearly showed that the use of face masks decreased the amount of inhaled allergen by about 90%. Rat allergen was also detected in dust collected from mattresses of laboratory animal workers (25).

**Table 4:** **Studies related to exposure assessment to rat allergen**.

Study	Environment/Country	Assay	Sampling method	Allergen level	
Hollander et al. (85)	Laboratory animal facilities, Netherlands	RUA	Air samples (personal)	Median	
		Sandwich ELISA	Job categories, *n* = 251		
		(pAB)	Animal caretaker, *n* = 90	1.6 ng/m^3^	
			Animal technicians, *n* = 87	0.77 ng/m^3^	
			Supervisor, *n* = 14	0.65 ng/m^3^	
			Scientists, *n* = 51	<LOD	
			Scientific assistant, *n* = 9	<LOD
			Task categories, *n* = 196	GM	Range
			Cage emptying, *n* = 29	5.6 ng/m^3^	<LOD–1600 ng/m^3^
			Changing animals, *n* = 35	5.3 ng/m^3^	<LOD–127 ng/m^3^
			Feeding animals, *n* = 20	2.4 ng/m^3^	<LOD–60 ng/m^3^
			Handling animals, *n* = 34	1.2 ng/m^3^	<LOD–94 ng/m^3^
			Experiments, *n* = 25	0.85 ng/m^3^	<LOD–9.0 ng/m^3^ s
			Biotechnical work, *n* = 23	0.83 ng/m^3^	<LOD–34 ng/m^3^
			Cage wash, *n* = 8	0.81 ng/m^3^	<LOD–4.9 ng/m^3^
			Cleaning rooms, *n* = 15	0.80 ng/m^3^	<LOD–14 ng/m^3^
Lieutier-Colas et al. (96)	Laboratory animal facilities, France	Rat n 1	Air samples	Range of GM	
		Sandwich ELISA	12 Facilities (personal), *n* = 113	0.49–48.96 ng/m^3^	
		(mAB)	12 Facilities (stationary), *n* = 128	0.43–27.36 ng/m^3^	
			Room categories (stationary)	AM	
			Rat rooms, *n* = 65	53.1 ng/m^3^	
			Experimental rooms, *n* = 56	9.7 ng/m^3^	
			Job categories (personal)	AM	
			Animal technicians, *n* = 27	72.3 ng/m^3^	
			Laboratory technicians, *n* = 26	21.2 ng/m^3^	
			Students, *n* = 33	33.7 ng/m^3^	
			Scientist, *n* = 27	24.0 ng/m^3^	
			Task categories (personal)		
			Cage cleaning and rat feeding, *n* = 34	91.1 ng/m^3^	
			Handling rats, *n* = 31	5.4 ng/m^3^	
Renström et al. (97)	Laboratory animal facility, Sweden	Rat n 1	Nasal air samplers	Median	IQR
		Sandwich ELISA	High exposure	
		(mAB)	No mask, *n* = 11	2.6 ng/h	0.6–5.0 ng/h
			P2-mask, *n* = 10	0.06 ng/h	<LOD–0.7 ng/h
			Low exposure	
			No mask, *n* = 25	0.1 ng/h	<LOD–0.2 ng/h
			P2-mask, *n* = 10	<LOD	<LOD– <LOD
Krop et al. (25)	Homes of laboratory animal workers, Netherlands	RUA	Reservoir dust from mattresses	GM	95% CI
		Sandwich ELISA	Laboratory animal workers, *n* = 15	39.3 ng/g	19.8–78.0 ng/g
		(pAB)	Controls (no contact to animals), *n* = 15	7.6 ng/g	4.7–12.2 ng/g
Perry et al. (80)	Homes (inner city), USA	Rat n 1	Reservoir dust from bed, furniture, floor	Median	Range
		Sandwich ELISA	Bedroom, *n* = 602	<LOD	<LOD–1413 ng/g
		(mAB)	Living room, *n* = 603	<LOD	<LOD–3380 ng/g
			Kitchen, *n* = 556	<LOD	<LOD–4620 ng/g

*RUA, rat urinary allergens; pAB, polyclonal antibodies; AM, arithmetic mean; GM, geometric mean; IQR, interquartile range; CI, confidence interval; LOD, limit of detection*.

In contrast to mice, the distribution of rat allergens outside of occupational settings is not well studied, probably due to much lower prevalence of rats indoors. Thus far, only one study has examined rat allergen exposure in the home environment (80) and reported Rat n 1 in 33% of inner city homes (settle dust samples). This is in contrast to mouse allergen, which was detectable in 95% of the homes. Rat allergen was more common in the TV/living room (27%) than in the kitchen (19%) and bedroom (21%). The median level for all rooms was below the limit of detection, and there was no correlation between rat and mouse allergens in dust samples. The authors attributed this dissimilarity to the differences in nesting habits between the both rodents. Rats do not nest in buildings, but rather build their nests in underground burrows in close proximity to water. Mice on the other hand are more likely to live indoors and nest near food stores. These territorial differences were compatible with reported rodent infestations, where 51% of families reported mice infestation, whereas only 8% reported problems with rats. Another possible explanation for the disparity between the prevalence of mouse and rat allergen levels is that the rat allergen assay was not sensitive enough to detect allergens in these samples. A second study focusing on allergen exposure in urban schools and homes assessed Rat n 1 concentrations in settled and airborne dust using the very sensitive MARIA technology (68). Again, no significant differences in rat allergen levels were detected in this sample set, suggesting that rat allergen exposure may occur primarily outdoors.

## Horse (*Equus Caballus*)

Horse allergy mainly affects people who are in direct contact to horses, either occupationally or for recreational purposes. Exposed subjects are farmers, stable-workers, breeders, veterinarians, and horse owners or riders. The prevalence of horse sensitization in occupational settings varies between 3.6 and 16.5% (98). However, the sensitization rate in the general population is not well known. Some cases of horse allergy, despite a lack of regular exposure, have been described in children (99) and adults (100). A retrospective study of horse allergy revealed a sensitization rate of 2.7% in a population of 23,460 children who underwent skin prick testing (101). A recent Italian multicenter study in an urban population with respiratory allergy reported that the prevalence of sensitization to horse dander was 5.38% among atopic subjects (102). Only 27% of horse-sensitized patients reported direct contact with the animals. Therefore, the authors of both studies recommended that the horse allergen be included in the standard panel for the diagnosis of respiratory allergy.

Several horse allergens have been identified and characterized, with Equ c 1 reported as the most important. Equ c 1 is found at high concentrations in dander and saliva, as well as in small quantities in urine (103). The 22-kDa glycoprotein belongs to the lipocalin family (104) and has surfactant properties (105). The other proteins, Equ c 2, lipocalin (16 kDa); Equ c 3, horse serum albumin (65 kDa); Equ c 4 (18.7 kDa); and Equ c 5 (16.7 kDa), both latherins, were classified as minor allergens (11, 50). The analysis of the allergenic composition of horse dander from several breeds showed considerable inter-breed and within-breed variations but no breed-specific allergens (106). Moreover, all dander extracts contained the most important allergens.

The dispersion of horse allergens have been studied in different environments, including stables and their immediate surroundings as well as in public places such as schools and day-care centers (Table 5). Most of these studies were performed in Sweden, where the rate of horse ownership is high and horseback riding has become increasingly popular. To detect horse allergen in environmental samples, a sandwich ELISA based on monoclonal antibodies was developed and established by Emenius et al. (107). The two monoclonal antibodies, 103 and 14G4, were produced against horse dander extract, and recognize different epitopes of the same molecule, but the target protein was not further characterized. Immunoblotting analysis demonstrated antibody binding to a protein of around 16 kDa (108). However, the horse allergen could not be identified because Equ c 2, Equ c 4, and Equ c 5 are within similar molecular weight range. Therefore, the ELISA assay is often referred to as Equ c x in the literature. In 2009, Emenius et al. (109) assumed that the antibodies recognize the allergen Equ c 4, but further identification is necessary. The horse allergen assay is in the meantime, commercially available and provided as Equ c 4 ELISA (Indoor Biotechnologies) with horse hair extract as the calibration standard. All the studies described below used this monoclonal assay with minor modifications (different standards and detection limits). Allergen concentrations are expressed as units per milliliter, where 1 unit is equal to 1 ng protein of the horse standard.

**Table 5:** **Studies related to exposure assessment to horse allergen**.

Study	Environment/country	Assay	Sampling method	Allergen level	
Emenius et al. (107)	Inside and outside a stable, Sweden	Equ c x	Air samples, *n* = 17	AM	
		Sandwich ELISA	Inside the stable	439000 U/m^3^	
		(mAB)	Stable entrance	1140 U/m^3^	
			10–20 m from stable	150 U/m^3^	
			40–500 m from stable	<LOD	
Elfman et al. (108)	Inside and outside a stable, Sweden	Equ c x	Air samples (stationary)	Median	Range
		Sandwich ELISA	Inside the stable, *n* = 12	4300 U/m^3^	1926–6272 U/m^3^
		(mAB)	Stable entrance, *n* = 10	316 U/m^3^	169–706 U/m^3^
			Source area, *n* = 49	16 U/m^3^	<LOD–203 U/m^3^
			50 m from stable, *n* = 27	<LOD	<LOD–41 U/m^3^
			100–500 m from stable, *n* = 91	<LOD	<LOD–24 U/m^3^
			Electrostatic cloths, *n* = 29	Median	Range
			Source area	27000 U/m^2^	3000–97000 U/m^2^
			25–50 m from stable	30000 U/m^2^	5000–195000 U/m^2^
			>100 m from stable	<LOD	<LOD–3000 kU/m^2^
Emenius et al. (109)	Homes nearby stables, vicinity of a horse track, Sweden	Equ c x	Petri dishes (14 days)	Positive samples *n*	
		Sandwich ELISA (mAB)	Indoors (living rooms), *n* = 45	6 (Apartments < 20 m from stable or families with horse contact)	
			Outdoors (balconies), *n* = 26	16 (15 Apartments < 250 m from stable)	
			Aspen leaves	Allergen level	
			1 m from horse track	100%	
			10 m from horse track	31–37%	
			25 m from horse track	8–9%	
			No horse track	None	
Kim et al. (110)	Schools, Sweden	Equ c x	Reservoir dust from desks, chairs and floor, *n* = 92	Median	Range
		Sandwich ELISA		945 U/g	<LOD–31000 U/g
		(mAB)			
Merrit et al. (113)	Schools, Sweden	Equ c x	Reservoir dust from furniture and floor	GM	
		Sandwich ELISA	Total, *n* = 116	1343 U/g	
		(mAB)	Classes > 12% horse contact	2051 U/g	
			Classes < 12% horse contact	880 U/g	
			Petri dishes (14 days)	GM
			Total, *n* = 116	73.9 U/m^2^/day	
			Classes > 12% horse contact	96.2 U/m^2^/day	
			Classes < 12% horse contact	65.7 U/m^2^/day	
Cai et al. (67)	Day-care centers, Sweden	Equ c x	Petri dishes (30–40 days)	GM	Range
		Sandwich ELISA	Diverse rooms, *n* = 97	5 U/m^2^/day	<LOD–208.7 U/m^2^/day
		(mAB)			

*mAB, monoclonal antibodies; GM, geometric mean; LOD, limit of detection; AM, arithmetic mean*.

The first study performed to detect horse allergen in the environment assessed airborne allergen levels at different distances from the stable (107). As expected, the highest allergen concentrations were measured inside the stable, and were approximately 500-fold higher than levels measured outdoors at the stable entrance. The levels of horse allergen declined rapidly with increasing distance from the stable and were not detected in air samples collected 40 m from the stable. A similar dispersal pattern was obtained with settled dust samples collected from hard surfaces wiped with compresses (allergen levels not stated). The dispersion of airborne horse allergen around the stable was also analyzed by Elfman et al. (108) during different seasons. The authors additionally investigated the influence of weather conditions such as temperature, relative and absolute humidity, wind speed and direction, and reported that horse allergen generally spread about 50 m from the stable and outdoor areas where horses were kept (e.g., pastures, riding grounds = source area). Depending on wind speed and direction, low levels of horse allergen (2–4 U/m^3^) were sometimes detected at distances up to 500 m from the source area. Allergen levels did not correlate to air temperature or humidity, but were influenced by the seasons; concentrations in winter were lower than in summer. At the stable entrance, the median level in summer was 316 and 123 U/m^3^ in winter, and in the source area 16 and 8.3 U/m^3^, respectively. More rain in autumn and a frozen ground in winter were mentioned as possible explanations for the reduced levels of airborne allergen. The rapid decrease in horse allergen with increasing distance from the stable was confirmed by analyzing settled dust collected with electrostatic cloths. All but one sample collected 100 m from the source area were below the detection limit.

Further research by Emenius et al. (109) examined the transfer of horse allergen into homes located near the stables. In apartments, Petri dishes were placed indoors (living rooms) or outdoors (balconies). Only 6 out of 45 indoor samples had detectable horse allergen levels (three families with horse contact) and 16 out of 26 outdoor samples were positive. Indoor levels were about 1–2% of the outdoor levels. In the second part of this study, the dispersion of allergens was investigated using a very unusual method. At different distances from a horse track, aspen leaves were collected and then extracted. The allergen level at 1 m from the track was set to 100%. At a distance of 25 m from the track, <10% of the original allergen concentration was found. In conclusion, horse allergens seem to disperse poorly through the air as allergen levels drop quickly with increasing distance from the source.

The presence of horse allergens in schools was first investigated by Kim et al. (110). Settled dust from desks, chairs, and floors was collected from 8 primary schools and 23 classrooms (*n* = 92) in Sweden. Horse allergens were found at high levels in most classrooms (median 945 U/g), and asthma and respiratory symptoms were more common in schools where higher levels of horse allergens were measured. No information on the prevalence of horse ownership in the families was available, but common horse contact was expected due to the geographical region (rural suburb of Uppsala). These data were used for two further projects to compare the school environment in China (111) and Korea (112) using the same sampling strategy (settled dust) and analysis method (Equ c × ELISA). In contrast to Swedish classrooms, none of the Chinese samples (*n* = 78, 39 classrooms) contained horse allergen. In Korea, horse allergen was only detected in one sample (*n* = 68, 34 classrooms).

The aim of another study was to investigate the correlation between horse allergen levels in schools and the number of children, who came into contact with horses in their leisure time (113). Petri dish and vacuumed dust samples were collected in 116 classrooms from 35 primary and secondary schools situated in inner-cities and in rural locations (county of Uppsala). In classrooms where many children had regular horse contact (>12%), the levels of horse allergen were significantly higher (for both sampling methods) than in classrooms where children reported less or no contact. Furthermore, weekly measurements with Petri dishes were performed in 20 classrooms during a 10-week period. The results showed that horse allergen levels were strongly variable during the 10-weeks and the authors recommended repeated measurements when assessing indoor allergen exposure. Horse allergen contamination was also common in Swedish day-care centers (67), where allergen was detected in 63% of Petri dish dust samples with geometric mean of 5 ng/m^2^/day.

## Cattle (*Bos Domesticus*)

Cattle allergy is almost exclusively associated with occupational exposure and occurs primarily in cattle farmers. Studies in Scandinavian countries found that 5–20% of farmers are sensitized to cattle allergens (114–116). Moreover, the German Cattle Allergy Study has indicated that 9.1% of 5627 farmers with occupational airway diseases were due to cattle allergies (117). The effect of the farming environment on sensitization to different allergens was investigated in children (118) and adults (119). For both, no significant differences were observed with respect to sensitization against most of the common allergens (e.g., house dust mites, pets, pollens). Only cattle sensitization was more prevalent among subjects living on farms compared with those not living on farms.

The main sources of bovine allergens are cow hair and dander, but allergens are also found in urine, saliva, whey, amniotic fluid, and beef (120). Early investigations of bovine materials have found 17 different antigenic components, several of which have been currently identified and characterized as allergens. The lipocalin Bos d 2 is the major respiratory allergen in cow dander (121, 122), with several isoforms in existence (123). It is produced in the sweat glands and transported to the skin surface as a carrier of pheromones (124). Other bovine allergens are: Bos d 3 (11 kDa), calcium-binding protein; Bos d 4 (14 kDa), α-lactoglobulin; Bos d 5 (18 kDa), β-lactoglobulin; Bos d 6 (67 kDa), serum albumin; Bos d 7 (150 kDa), immunoglobulin; and Bos d 8 (19–25 kDa), caseins (11, 50). Bos d 4, 5, and 8 are major allergens in cow’s milk and play an important role in food allergy (125).

Measurements of epithelial bovine allergens have been carried out mainly in occupational settings, stables and homes of farmers (Table 6). To quantify allergen concentrations, several different methods have been developed including: (1) ELISA inhibition with polyclonal antibodies against bovine skin scrape (126), (2) Rocket immunoelectrophoresis with rabbit antiserum against the major allergen Bos d 2 (127), (3) sandwich ELISA with polyclonal antibodies against an extract prepared from hair of different cattle breeds (128), and (4) sandwich ELISA with anti-Bos d 2 monoclonal antibodies 3D4 and mAB1 (129). Recently, the last method with recombinant Bos d 2 as a reference standard was made commercially available (Indoor Biotechnologies).

**Table 6:** **Studies related to exposure assessment to cattle allergens**.

Study	Environment/country	Assay	Sampling method	Allergen level
Virtanen et al. (130)	Stables, Finland	Bovine epithelial antigen	Air samples (stationary)	AM	Range
		ELISA inhibition	Feeding passage, *n* = 18	350 ng/m^3^	40–2700 ng/m^3^
		(pAB)	Manure passage, *n* = 18	730 ng/m^3^	40–9500 ng/m^3^
Ylönen et al. (129)	Stables, Finland	Bos d 2 Sandwich ELISA (mAB)	Air samples (stationary)	AM	Range
			Stables, *n* = 19	280 ng/m^3^	54–804 ng/m^3^
Hinze et al. (127)	Homes of farmers, Germany	Bos d 2 Rocket immunelectrophoresis (pAB)	Reservoir dust from floor	AM	
			Barn and living quarters		
			Separated		
			Corridor, *n* = 17	40.6 μg/g	
			Living room, *n* = 17	82.4 μg/g	
			Bedroom, *n* = 16	56.3 μg/g	
			In the same building		
			Corridor, *n* = 13	103.6 μg/g	
			Living room, *n* = 13	112.1 μg/g	
			Bedroom, *n* = 13	150.4 μg/g	
Berger et al. (131)	Stables and homes of farmers (three groups with different cattle exposure), Germany	Bos d 2 Rocket immunelectrophoresis	Reservoir dust	Median	Range
		(pAB)	Stables, *n* = 36	20,400 μg/g	680–55,400 μg/g
			Homes (living room floor)	
			Former contact, *n* = 10	13 μg/g	3–43 μg/g
			Indirect contact, *n* = 13	148 μg/g	34–2929 μg/g
			Direct contact, *n* = 23	316 μg/g	46–4209 μg/g
			Homes (mattress)	
			Former contact, *n* = 10	12 μg/g	4–381 μg/g
			Indirect contact, *n* = 13	195 μg/g	15–403μg/g
			Direct contact, *n* = 23	265 μg/g	31–1268 μg/g
Zahradnik et al. (128)	Stables and homes of farmers and controls, Germany	Cow hair allergen	Electrostatic cloths (14 days)	Median	Range
		Sandwich ELISA	Stables	
		(pAB)	Cow, *n* = 37	51,700 μg/m^2^	4760–559,000 μg/m^2^
			Goat, *n* = 6	315.7 μg/m^2^	91–701.4 μg/m^2^
			Other, *n* = 14	1.2 μg/m^2^	<LOD–6.5 μg/m^2^
			Homes	
			Cattle farmers, *n* = 128	22.6 μg/m^2^	0.3–900 μg/m^2^
			Urban dwellers, *n* = 32	0.2 μg/m^2^	<LOD–2.7 μg/m^2^
Williams et al. (132)	Inside and outside of homes nearby dairy facilities, USA	Bos d 2	Air samples (stationary)	Median	Maximum
		Sandwich ELISA	Indoor	
		(mAB)	Proximal, *n* = 16	0.12 μg/m^3^	0.97 μg/m^3^
			Intermediate, *n* = 5	0.01 μg/m^3^	0.12 μg/m^3^
			Distal, *n* = 12	0.01 μg/m^3^	0.03 μg/m^3^
			Outdoor	
			Proximal, *n* = 19	0.66 μg/m^3^	1.87 μg/m^3^
			Intermediate, *n* = 6	0.17 μg/m^3^	0.29 μg/m^3^
			Distal, *n* = 12	0.01 μg/m^3^	0.10 μg/m^3^

*mAB, monoclonal antibodies; pAB, polyclonal antibodies; LOD, limit of detection; AM, arithmetic mean*.

In cow stables, levels of bovine allergen were estimated in airborne dust (129, 130) as well as in settled dust samples (128, 131). All studies reported very high and strongly variable allergen concentrations. Allergen levels differed up to 200-fold between stables and were about 1000-fold higher than in homes. Virtanen et al. (133) examined the long-term variability in airborne allergen concentrations and found that in some cow stables, bovine allergen levels tend to be low, whereas in others the levels are consistently high. These variations are likely explained by factors associated with stable characteristics, such as size, heating, ventilation, and construction details of the building. Measurements of bovine allergens were also performed in stables of other animals (128). Whereas, only trace amounts of allergens were detectable in horse, sheep, pig, and chicken stables, goat stables had slightly increased allergen levels, most likely due to cross-reactivity between cow and goat epithelia.

The presence of cattle allergens in homes of dairy farmers was initially investigated by Hinze et al. (127). Floor dust samples were collected in different rooms of homes from patients with cow hair asthma and analyzed for Bos d 2 using Rocket immunelectrophoresis. The quantities of Bos d 2 detected were dependent on the architectural setup and floor cover. The separation of barn and living quarters (not in the same building) led to a marked reduction in Bos d 2 levels in house dust. Bos d 2 concentrations were also considerably lower in carpets than on tiles or linoleum. Furthermore, high indoor Bos d 2 levels were shown to correlate with the degree of IgE sensitization.

Using the same analytical methods, allergen levels in homes were analyzed in relation to the exposure intensity of cattle farmers (131). Farmers with occupational asthma or rhinitis caused by cow dander were divided into three groups: (1) no contact with cattle (giving-up the cattle husbandry for at least 2 years), (2) indirect exposure through family members, and (3) regular contact with cattle. The results showed a highly significant association between level of exposure and level of allergen. The terminating or limiting contact to cows reduced Bos d 2 concentrations in both living room and mattress dust. The aim of a further study was to assess bovine allergen exposure in homes of cattle farmers by sampling settled airborne dust using electrostatic cloths (128). Cow hair allergens measured by polyclonal antibody-based ELISA were detected with a wide variation among the individual samples (0.3–900 μg/m^2^). The results, categorized by room type, showed significantly higher allergen concentrations in changing rooms compared with living rooms, bedrooms, or kitchens. As a control, dust sampling was also performed in urban dwellings. Interestingly, although none of the household members had any contact with cattle farms, the majority of urban samples were positive in the assay, though at very low concentrations. The median of 0.2 μg/m^2^ was 100-fold lower in comparison to farmer homes. Because, the dispersal of cattle allergens from rural to urban environments through the ambient air was quite implausible, the authors supposed that the positive results were caused by the cross-reactivity between human and pet hair or by the presence of bovine allergens derived from foods such as milk and beef. Follow-up analysis using monoclonal antibody-based ELISA (Indoor Biotechnologies) confirmed the presence of the major respiratory allergen Bos d 2 in these dust samples and extracts from foods [(134), EAACI abstract].

Finally, the distribution of cattle allergens was assessed at different distances to dairy facilities (132). The study was conducted in the Yakima Valley, Washington State, USA, where over 60 industrial scale dairies operate. Airborne samples were collected inside and outside homes and analyzed using Bos d 2 ELISA (Indoor Biotechnologies). Homes with resident dairy facility workers or cows on the premises were excluded to minimize the influence of occupational exposures on indoor environments. Similar to studies of dispersion of horse allergen, an allergen concentration gradient was observed. Outdoor and indoor results for airborne Bos d 2 showed the highest concentrations at proximal homes closest to dairies (within a 1/4 mile, 0.4 km), and lowest concentrations in distal homes farthest from dairies (>3 miles, 4.8 km). Median outdoor levels of Bos d 2 were significantly higher at proximal and intermediate homes compared with indoor levels.

## Summary

Measurements of animal allergens have been extensively performed during the past few decades. Allergen exposure to animal allergens occurs in a wide range of indoor environments including homes, educational facilities, workplaces, and different kind of public buildings and modes of public transportation. Mostly, settled dust and airborne dust samples were collected to measure animal allergen levels. The variability of allergen concentrations in a particular environment is high and dependent on numerous factors, the most important being the presence of animals. Highest allergen levels have generally been found in homes with pets, laboratory animal facilities housing mice or rats, and cow or horse stables. However, high allergen levels have also been frequently detected in locations where no animals reside (e.g., schools and public places), most likely due to passive transfer via human clothing or hair. Some studies have demonstrated that animal allergen levels in these mostly public environments can be significantly higher than in domestic areas without animals. The number of pet owners is one of the strongest predictors of increased allergen levels in these settings. Apart from the presence of animals or number of individuals with direct and frequent contact to animals, differences in allergen concentrations are associated with various building-related factors such as size and type of room, type of flooring, and furniture, cleaning frequency, ventilation system, and also the distance to animal rooms or stables. For example, carpets, mattresses, and upholstery are consistently found to have much higher concentrations of animal allergens than smooth surfaces. Allergen levels can also vary in different parts of the world, which appears to be primarily influenced by regional and cultural differences in pet ownership or livestock farming. Rodent infestation is another factor that is strongly associated with increased mouse allergen levels. In contrast to mites, mammalian allergens seem to be independent of climate or seasonal variations.

Besides environmental factors, sample collection strategies and analytic methods enormously influence the results of exposure measurements. A variety of commercially available and well-characterized sampling equipment have been used in the studies. For each sampling method, differences exist regarding features, such as sampling pump, air flow rate, vacuum power, collection device, filter type, sampling duration and number, and size and type of surfaces sampled. Concerning the ELISA method, which was used to quantify allergen concentrations, the variations comprise the type of antibodies, calibration standard and its protein determination, replicate precision, and detection/visualization methods. Differences in data analysis for example, median, arithmetic, or geometric mean and calculation of the results as nanograms per gram or nanograms per meter square impede the direct comparison of the data produced in different studies.

## Conclusion and Recommendations

Environmental allergen exposure plays a significant role in the development of asthma and allergy. Allergic diseases are important public health concerns because of severity of symptoms, reduced quality of life, and limited productivity of the affected persons and high healthcare costs. Therefore, the identification of major sites of exposure and factors influencing allergen levels is essential to prevent allergic health effects. Specific measures to reduce or to avoid exposure to allergens can be initiated. Moreover, the knowledge of exposure levels is helpful to estimate the risk of sensitization or induction of symptoms in occupational or environmental settings. There is still lack of information on risk limits. One reason is the complexity of allergen monitoring, which is a multi-step task requiring various tools and techniques. Although, more allergen exposure data and more accurate methods are becoming available, a general standardization of sampling, and analytical procedures is much needed. The development of consensus protocols can be advantageous in the future for a better comparison of data from different studies. In the case of animal allergens, one basic requirement for standardization is fulfilled through commercial availability of monoclonal sandwich ELISA/MARIA kits for the detection of major allergens. Some assays, e.g., Fel d 1 and Can f 1 ELISA, have already reached global dissemination.

Apart from methodological issues, the estimation of “general” risk levels is complex. In contrast to toxic substances, which affect more or less all exposed individuals, the reactivity to the same allergen can vary extremely between people. Some persons will never become sensitized even at high exposure. Allergen levels associated with an increased risk of disease and/or sensitization are certainly different for healthy, sensitized and allergic persons. Finally, the determination of risk levels should also include the type of environment (workplace, home, school) because the circumstances of personal exposure are different. Therefore, a clear definition of strategy which provides the best proxy of allergen burden for different exposure scenarios is needed. The definition should include the type of dust, dust sampling procedure with validated protocol, type of allergenic substances (allergen mix or single allergen) and standardized immunoassay.

## Conflict of Interest Statement

The authors declare that the research was conducted in the absence of any commercial or financial relationships that could be construed as a potential conflict of interest.
